# Therapeutic Targeting of Krüppel-Like Factor 4 and Its Pharmacological Potential in Parkinson’s Disease: a Comprehensive Review

**DOI:** 10.1007/s12035-023-03800-2

**Published:** 2023-11-24

**Authors:** Mohammad Yasin Zamanian, Maryam Golmohammadi, Rana Sherdil Amin, Ghadeer Sabah Bustani, Rosario Mireya Romero-Parra, Rahman S. Zabibah, Tuba Oz, Abduladheem Turki Jalil, Afsaneh Soltani, Małgorzata Kujawska

**Affiliations:** 1 Neurophysiology Research Center, Hamadan University of Medical Sciences, Hamadan, 6718773654 Iran; 2 Department of Pharmacology and Toxicology, School of Pharmacy, Hamadan University of Medical Sciences, Hamadan, 6718773654 Iran; 3 School of Medicine, Shahid Beheshti University of Medical Sciences, Tehran, 1988873554 Iran; 4 Sharif Medical & Dental College, Lahore, Pakistan; 5 College of Dentistry, The Islamic University, Najaf, Iraq; 6 General Studies, Universidad Continental, Lima, Perú; 7 Medical Laboratory Technology Department, College of Medical Technology, The Islamic University, Najaf, Iraq; 8 Department of Toxicology, Poznan University of Medical Sciences, Rokietnicka 3, 60-806 Poznan, Poland; 9 Medical Laboratories Techniques Department, Al-Mustaqbal University College, Babylon, Hilla, 51001 Iraq

**Keywords:** Parkinson’s disease, Neuroinflammation, KLF4, Apoptosis, Oxidative stress, Autophagy

## Abstract

Krüppel-like factor 4 (KLF4), a zinc finger transcription factor, is found in different human tissues and shows diverse regulatory activities in a cell-dependent manner. In the brain, KLF4 controls various neurophysiological and neuropathological processes, and its contribution to various neurological diseases has been widely reported. Parkinson’s disease (PD) is an age-related neurodegenerative disease that might have a connection with KLF4. In this review, we discussed the potential implication of KLF4 in fundamental molecular mechanisms of PD, including aberrant proteostasis, neuroinflammation, apoptosis, oxidative stress, and iron overload. The evidence collected herein sheds new light on KLF4-mediated pathways, which manipulation appears to be a promising therapeutic target for PD management. However, there is a gap in the knowledge on this topic, and extended research is required to understand the translational value of the KLF4-oriented therapeutical approach in PD.

## Introduction

Kruppel-like factor 4 (KLF4), a zinc finger transcription family member, was originally isolated from an NIH3T3 cDNA library in 1996 [1, 2]. KLF4 is a highly conserved factor found in many organisms, from zebrafish to humans, and it is expressed in numerous human tissues, such as intestinal epithelial cells, skin, and neural stem cells [3–5]. KLF4 plays a significant role in controlling multiple biological pathways, including proliferation, embryogenesis, differentiation, neuroinflammation, oxidative stress, and apoptosis [6–10]. In the CNS, KLF4 controls the proliferation, migration, and differentiation of neural stem cells (NSCs), axon regeneration, and migration of radial neurons by regulating STAT3 [11, 12]. KLF4 downregulation is critical for neural differentiation, and overexpression of KLF4 in NSCs impedes their proliferation and differentiation. Moreover, KLF4 has a higher expression in the embryonic brain, where it is involved in the self-renewal of embryonic stem cells and is gradually downregulated postnatally. In this regard, KLF4 dysregulation may lead to hydrocephalus [13]. Additionally, overexpression of KLF4 in the endothelial cells of the microvasculature in the CNS can result in the development of cerebral cavernous malformation [14]. Recently, the protective role of KLF4 via the Nrf2/Trx1 downstream pathway against the blood–brain barrier destruction after cerebral ischemia–reperfusion was demonstrated [15]. These findings align with the context-dependent function KLF4. Interestingly, a review of the literature by Cheng et al. indicates the implication of KLF4 to different neurological diseases like Parkinson’s disease (PD), Alzheimer’s disease (AD), epilepsy, schizophrenia, and hydrocephalus making it a potential therapeutic target for their management [16]. In neurodegenerative diseases, KLF4 has been found to regulate neuroinflammation, neuronal apoptosis, axon regeneration, and iron accumulation, all of which are relevant to the pathogenesis of AD [17, 18].

Huntington’s disease (HD) is a progressive neurological condition that arises from an abnormal increase in the number of CAG repeats within the huntingtin gene [19]. KLF4 is a reprogramming factor that is utilized in the generation of iPSCs from fibroblasts or peripheral blood mononuclear cells of individuals with HD [20]. iPSCs have been generated from somatic cells of HD patients and can serve as a valuable tool for studying the disease and developing potential therapies [20–22]. Furthermore, KLF4 is mentioned as one of the endogenous pluripotency-associated markers that are reactivated and expressed in the HD-iPSC lines.

PD is a progressive neurodegenerative disease with an alarming negative impact on individuals and healthcare systems in developed and developing countries [23, 24]. The current understanding of the underlying mechanisms of PD is mainly focused on the progressive degeneration of dopaminergic neurons in the substantia nigra, leading to a decrease in dopamine levels in the brain. Subsequently, it results in cardinal motor symptoms, including tremors, rigidity, balance issues, and loss of spontaneous movement [25, 26]. Despite the clear hallmarks of PD, the etiology of the disease is still obscure. The most prominent pathological feature is the aggregation of normal and abnormal strains of a presynaptic protein α-synuclein. The oligomeric forms of α-synuclein, which trigger inflammation and cause the death of neurons, are the most toxic species [27]. There is strong evidence that these α-synuclein inclusions are cell-to-cell propagated in a prion-like manner [28]. Microglia-mediated neuroinflammation is another critical PD hallmark for its pathogenesis [29]. In addition, oxidative stress, iron overload, mitochondrial dysfunction, impaired autophagy, and apoptosis contribute to the progressive degeneration of dopaminergic neurons in PD [30–33]. A review of the literature on this area found that KLF4 is associated with neurodegenerative diseases, including PD. Given that KLF4 regulates neuron apoptosis, synaptic regeneration, oxidative stress, autophagy, and neuroinflammatory responses, the knowledge about the relationship between KLF4 and PD pathogenesis may enable the identification of novel targets for PD treatment [16]. PD etiology and pathological heterogeneity require a variety of models replicating different aspects of PD in animals to understand the critical pathomechanisms of the disease. Most of them are based on the environmental contribution to the pathological features of PD through various neurotoxins.

MPTP and its metabolite MPP + , rotenone (ROT), and 6-OHDA are commonly used to induce parkinsonian traits and recapitulate the key pathological hallmarks of PD in animal models [34]. Significantly, these neurotoxin-based models have been demonstrated to stimulate KLF4 expression [35–38], which suggests its potential contribution to the pathogenesis of PD, thus making KLF4 a potential target in the battle against PD.

In this review, we critically discussed a few available data on the controlling role of KLF4 in autophagy, neuronal apoptosis, oxidative stress, neuroinflammation, and other related mechanisms to show the relation between KLF_4_ and the pathogenesis of PD, which may underlie the cellular and molecular hallmarks of the disease. Overall, the involvement of KLF4 in neuroprotection and antioxidant defense suggests its potential as a therapeutic target for PD. Further research is needed to elucidate the precise mechanisms by which KLF4 exerts its neuroprotective effects and to explore its therapeutic potential in PD models and clinical settings.

## Overview of KLF4

Shields et al. identified and characterized the nuclear transcription factor KLF4 in 1996 [1]. KLF4 was first recognized in the intestine thereafter, and it turned out that this transcription factor was expressed in different human tissues and organs [3–5]. KLF4 is a reprogramming factor directly connected to the Sp/Klf family, a group of 26 transcription factors essential for cellular pathways [39–42]. KLF4 is a highly conserved gene across all vertebrate lineage. In humans, the gene was detected on chromosome 9q31, which possesses a 6.3 kb chromosome region and contains five exons [43–45]. In mice, KLF4 was found on chromosome 4, which covers the 3.5 kb region detected by northern blot analysis [46]. The structure of the carboxy-terminal of KLF4 has DNA binding sites within Cys2His2 (C2H2) zinc finger motifs, which modulate transcription through binding to CACCC-box and GC-rich elements [40, 47]. On the other hand, the amino-terminal of KLF4 has a highly variable regulatory site for transcription and contains both the transactivation domain as well as repression domain that justifies its dual function [43, 48]. KLF4 acts as a complex, versatile transcription factor that switches between different modes, including trans activator, trans repressor, oncogene, and tumor suppressor. It also regulates adaptive responses to various intracellular and extracellular stresses, like environmental changes, DNA damage, and oxidative stress [7, 49–52]. KLF4 is responsible for numerous cellular and biological processes via inducing cell cycle arrest, genomic stability, growth arrest, stem cell self-renewal, and apoptosis [53].

**Fig. 1:**
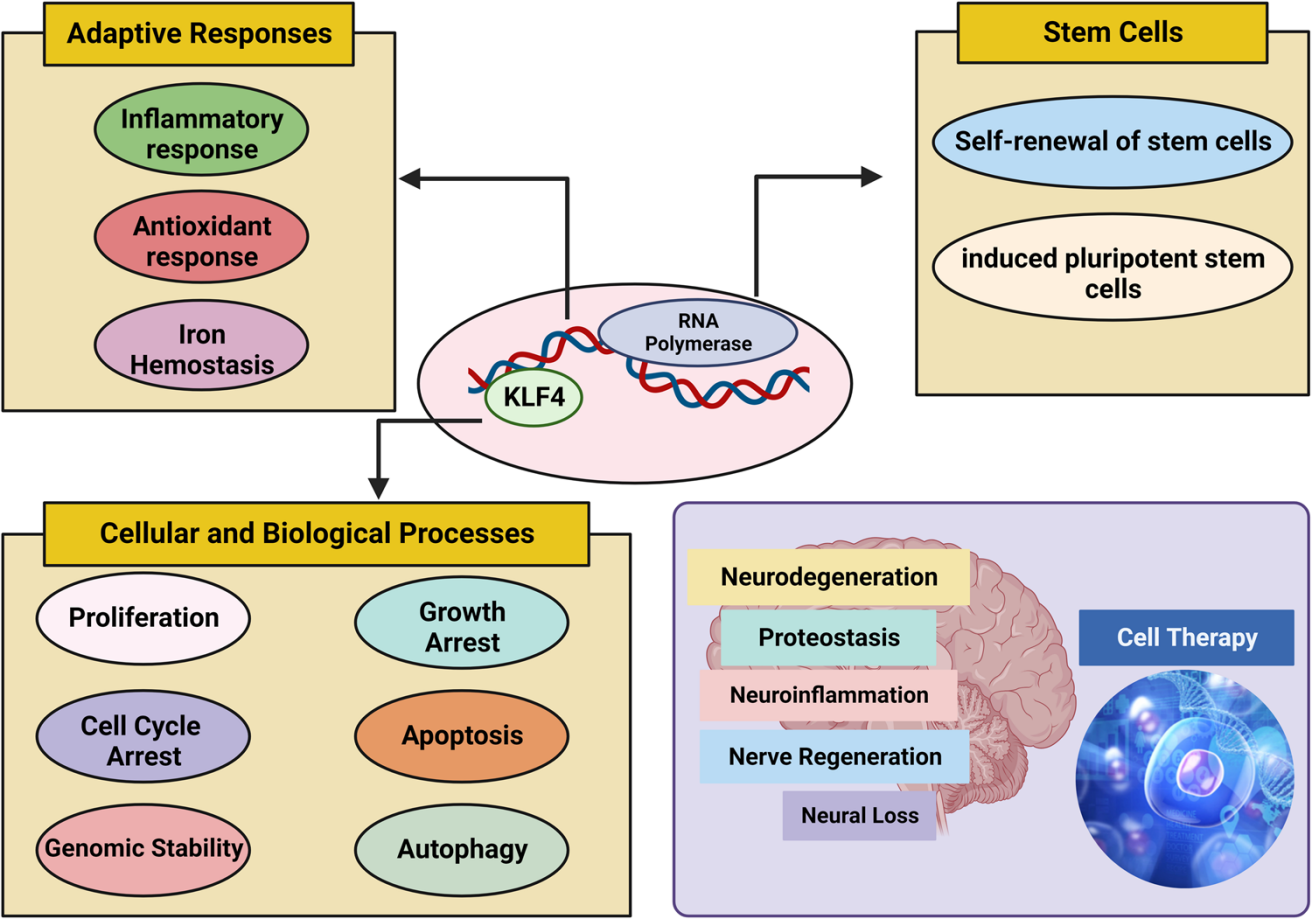
Multifunctionality of KLF4 and its implication to neurodegeneration management. Neuroinflammation plays a crucial role in the development of PD. It refers to the inflammation that occurs in the central nervous system as a result of molecules released from immune cells present in the brain or circulating in the blood. KLF4 plays a role in controlling oxidative stress and apoptosis in PD. It promotes oxidative stress and apoptosis, potentially through the inhibition of antioxidant enzymes

Reports depicted a high expression of KLF4 in neural stem cells and different parts of the brain, like the hypothalamus, hippocampus, and cerebral cortex [11, 13, 18, 54, 55]. Many studies demonstrated KLF4 regulatory role in neuronal apoptosis, neuroinflammation, and nerve regeneration [17, 18, 55, 56]. KLF4 is involved in the regulation of neuroinflammation in AD. Neuroinflammation, a key factor in the development of AD, involves the activation of glial cells and the release of pro-inflammatory cytokines. Research has shown that there is a direct relationship between the expression of KLF4 and the occurrence of neuroinflammation induced by Aβ42. Oligomeric Aβ42 can increase KLF4 expression in microglial BV2 cells, mediated by activated P53 [17, 18]. Furthermore, in a study by Ahn et al., KLF4 expression was examined in the spinal cords of rats with EAE. The authors found increased KLF4 expression in the spinal cords of EAE rats compared to control rats. This suggests that KLF4 may play a role in the inflammatory response in the CNS during EAE and potentially in MS [57]. Chahoki et al. reported that the dysregulation of KLF4 and its correlation with miR-25-3p in EAE indicates a potential role for KLF4 in the pathogenesis of MS and highlights the complex interplay between transcription factors and microRNAs in autoimmune diseases. Additionally, KLF4 is involved in T cell development and has been shown to regulate Th17 cell differentiation, which plays a pathogenic role in EAE [58]. Additionally, Moore and colleagues demonstrated that the decrease in KLF4 expression led to the growth of axons in retinal ganglion cells following injury to the optic nerve [59]. Given the multifunctionality of KLF4 within CNS, its implication for the management of neurodegenerative disease can be considered (Fig. 1).

## Relevance of KLF4 in Parkinson’s Disease

### Role of KLF4 in Neuroinflammation

Neuroinflammation is a key contributor to the development and progression of PD [60, 61]. It is characterized by the activation of microglia and the release of pro-inflammatory molecules [62]. Chronic inflammation plays a role in the deterioration of dopaminergic neurons in the substantia nigra, which is responsible for the motor symptoms observed in PD [63]. Microglia can polarize into two phenotypes: M1 and M2 [64]. M1 microglia have a pro-inflammatory phenotype and release cytokines such as IL-1β and iNOS, which contribute to neuronal damage [65]. On the other hand, M2 microglia have an anti-inflammatory phenotype and release cytokines such as arginase-1 and CD163, which promote tissue repair and neuroprotection [66]. KLF4 has been recognized as a critical factor in controlling the polarization of microglial cells towards the M2 phenotype [67]. It promotes the expression of M2 markers, such as arginase-1, and inhibits the expression of M1 markers, such as iNOS and pro-inflammatory cytokines [68]. This shift towards the M2 phenotype is associated with a reduction in neuroinflammation and an improvement in neuronal survival. The activation of the PI3K/AKT pathway plays a vital role in limiting pro-inflammatory responses and promoting anti-inflammatory responses in macrophages [69]. The PI3K/AKT pathway is one of the signaling pathways that regulates the activation of KLF4 in microglia [70]. Activation of the PI3K/AKT pathway phosphorylates and activates AKT, which further activates KLF4 [71]. This suggests that the PI3K/AKT/KLF4 signaling axis plays a crucial role in regulating microglial polarization and neuroinflammation. In the study of El-Deeb et al., Flibanserin (Flib), a 5HT1A receptor modulator, was found to activate the PI3K/AKT pathway and induce the expression of KLF4 in a rat model of PD induced by Rotenone. The administration of Flib led to the activation of KLF4, which caused a transformation of microglial polarization from the inflammatory M1 phenotype to the anti-inflammatory M2 phenotype. This shift was associated with a reduction in pro-inflammatory markers and an improvement in motor function and neuronal integrity [72]. Overall, KLF4 plays a crucial role in regulating neuroinflammation by modulating microglial polarization. Activation of KLF4 promotes the M2 phenotype, which has anti-inflammatory and neuroprotective effects. Targeting the PI3K/AKT/KLF4 signaling axis may have therapeutic potential for neuroinflammatory diseases, including Parkinson’s disease.

When microglial cells are stimulated with lipopolysaccharide (LPS), KLF4 expression increases, and it translocates to the nucleus [73]. In the nucleus, KLF4 interacts with specific promoter elements of pro-inflammatory cytokine genes, such as TNF-α, MCP-1, and IL-6, and upregulates their expression [74, 75]. This leads to increased production of these cytokines, which are known to play a key role in neuroinflammation [73]. Additionally, KLF4 is also involved in the upregulation of iNOS and Cox-2, which are enzymes involved in the inflammatory response [73, 76]. Knockdown of KLF4 in microglial cells results in decreased expression of iNOS and Cox-2, leading to a decrease in NO production [77]. Kaushik et al. showed that the expression of the transcription factor Klf4 increased in BV-2 microglial cells in response to LPS treatment. The increase in KLF4 expression was dose-dependent and time-dependent, with higher doses of LPS and longer treatment times leading to higher levels of KLF4 expression. They also found that LPS treatment induced the expression of inflammatory mediators, including iNOS, Cox-2, and pro-inflammatory cytokines, in BV-2 cells. Furthermore, they demonstrated that KLF4 knockdown resulted in decreased expression of iNOS and reduced production of NO, indicating the role of KLF4 in mediating inflammation. Overall, these results suggest that KLF4 plays an important role in the inflammatory response of microglial cells to LPS stimulation [73]. In addition, KLF4 has been implicated in the regulation of inflammatory mediators, such as TNF-α and IL-6, which are known to contribute to neuroinflammation [78]. Furthermore, KLF4 has been shown to modulate the expression of genes involved in the inflammatory response.

miR-214-3p is a microRNA that has been implicated in various human diseases, including PD [79]. Zhou and colleagues demonstrated that in models of PD, the expression of SNHG14 was increased, while the expression of miR-214-3p was decreased. The knockdown of SNHG14 alleviated MPP^+^-induced damage in SK-N-SH cells by increasing cell viability, reducing cell apoptosis, and suppressing pro-inflammatory cytokine production. In terms of its mechanism, SNHG14 directly binds to and sequesters miR-214-3p, resulting in an increase in the levels of miR-214-3p. Subsequently, miR-214-3p interacts directly with the 3′ untranslated region (3′UTR) of KLF4 and reduces its expression. The silencing of SNHG14 in SK-N-SH cells provides protection against MPP + -induced cytotoxicity by upregulating miR-214-3p and subsequently downregulating KLF4 expression. In this study, the levels of TNF-α, IL-6, and IL-1β were evaluated in MPP^+^-stimulated SK-N-SH cells. This suggests that SNHG14 may play a role in regulating neuroinflammation by modulating the release of TNF-α and IL-6 2. The findings support the potential therapeutic targeting of SNHG14 in PD to alleviate neuroinflammation [80].

BIG1, also known as Brefeldin A-inhibited guanine nucleotide-exchange factor 1 (ARFGEF1), is a protein involved in cellular processes such as cell migration, neurite outgrowth, and regulation of inflammatory cytokines [81, 82]. You et al. showed that BIG1 plays a role in LPS-mediated neuroinflammation and migration in BV2 cells. The inhibition of BIG1 resulted in a decrease in the levels of pro-inflammatory cytokines (such as TNF-a, IL-1b, IL-6) and an increase in the levels of the anti-inflammatory cytokine IL-10. BIG1 knockdown also inhibited the production of NO and the expression of iNOS and COX-2. Furthermore, BIG1 knockdown suppressed cell migration in the presence of LPS. On the other hand, overexpression of BIG1 showed the opposite effects, promoting neuroinflammation and cell migration. Mechanistically, it was found that BIG1 regulated the PI3K/Akt/NF-kB signaling pathway, and the activation of this pathway reversed the protective effects of BIG1 knockdown. Furthermore, the research revealed that KLF4 played a role in the transcriptional regulation of BIG1 and that the overexpression of BIG1 reversed the effects of KLF4 knockdown in terms of neuroinflammation and migration. In summary, the potential therapeutic implications of targeting BIG1 in neurodegenerative diseases, specifically those associated with neuroinflammation like PD, include the reduction of pro-inflammatory cytokine production, inhibition of cell migration, and modulation of the PI3K/Akt/NF-κB signaling pathway. However, more research is required to fully comprehend the effectiveness of targeting BIG1 as a therapeutic strategy in these diseases [83].

### Role of KLF4 in Oxidative Stress

Oxidative stress is a crucial factor in the development of PD [24]. Oxidative stress is a condition characterized by an unequal balance between the generation of ROS and the capacity of the body’s antioxidant defense mechanisms to counteract them [84]. It contributes to the degeneration of dopaminergic neurons through various mechanisms, including mitochondrial dysfunction, neuroinflammation, and the oxidation of dopamine [85].

In PD, increased levels of ROS and oxidative stress can lead to damage to mitochondrial lipids, proteins, and DNA, impairing mitochondrial function and leading to cell and tissue damage [85]. Studies have also shown that genetic mutations associated with PD, such as those in the Parkin, PINK1, DJ-1, SNCA, LRRK2, and GBA genes, result in mitochondrial dysfunction and increased oxidative stress [86–88]. Moreover, various animal models of PD, including those induced by neurotoxins and genetic modifications, have provided evidence for the role of oxidative stress in the initiation and advancement of the disease [89].

SOD1 is an important enzyme that plays a critical role in protecting against oxidative stress [90]. It is the most important enzyme in various tissues, including neuronal cells, and helps to prevent cell damage and apoptosis [91]. In their study, Chen et al. discovered that the presence of the transcription factor KLF4 contributes to the neurotoxicity caused by MPP + . They observed that the expression of KLF4 increased over time and with higher doses of MPP + in M17 cells. Moreover, when KLF4 was overexpressed, it intensified the neurotoxic effects of MPP + , resulting in heightened vulnerability of the cells and increased oxidative stress. In contrast, inhibiting the expression of KLF4 reduced the harmful effects of MPP + . The study also demonstrated that the suppression of SOD1 by KLF4 played a crucial role in the development of oxidative stress and neurotoxicity caused by MPP + . Overexpression of KLF4 resulted in reduced SOD1 expression, while knockdown of KLF4 increased SOD1 expression. Additionally, KLF4 overexpression promotes cell vulnerability and LDH release, indicating increased oxidative damage [92]. These findings suggest that KLF4 may be a potential therapeutic target for the treatment of PD.

Aprepitant is a selective NK1 receptor antagonist that has shown potential therapeutic effects in various neurological diseases, including PD [93, 94]. The NK1 is a G-protein coupled receptor that is involved in various physiological and pathological processes. It is primarily activated by the substance P (SP), a neuropeptide that plays a role in neuroinflammation and neurodegenerative disorders [95, 96]. ERK5 is a member of the mitogen-activated protein kinase (MAPK) family [97]. It is involved in essential cellular functions such as cell growth, differentiation, and survival [98]. El-Deeb et al. showed that treatment with aprepitant had a neuroprotective effect in a rotenone-induced PD model. aprepitant improved motor function and reduced behavioral and motor disturbances in the rats. It also decreased striatal and substantia nigra neuronal cell death and preserved tyrosine hydroxylase (TH) immunoreactivity, which is indicative of dopaminergic neuron viability. Histopathological examination revealed that aprepitant treatment resulted in a higher count of intact neurons and milder glial cell infiltrates compared to the rotenone group. Immunohistochemical examination showed that aprepitant increased TH immunoreactivity and the count of TH-positive neurons in the substantia nigra region. Furthermore, aprepitant modulated the ERK5/KLF4 signaling pathway. It increased the levels of phosphorylated ERK5 (pERK5) and KLF4. These effects were associated with improvements in oxidative stress markers, such as increased reduced GSH levels and decreased MDA levels. In summary, KLF4 plays a role in regulating oxidative stress through the induction of antioxidant enzymes. Aprepitant, through the activation of ERK5/KLF4 signaling, can modulate oxidative stress and autophagy, leading to neuroprotective effects in PD models [38].

### Role of KLF4 in Apoptosis

Apoptosis, which refers to the process of programmed cell death, has a significant impact on PD [99]. Apoptotic cell death of DArgic (DA) neurons is a key feature of PD [99]. The selective loss of these neurons leads to the motor symptoms associated with the disease [99]. Various factors, such as mitochondrial dysfunction, oxidative stress, and protein misfolding, contribute to the activation of apoptotic pathways in DA neurons [100, 101]. Autoimmune cells, such as T cells and B cells, have been suggested to play a role in the development of PD [102]. These cells can recognize α-synuclein, a protein associated with PD, and promote neuroinflammation, leading to the apoptosis of DA neurons [102]. Apoptosis in PD involves the activation of initiator caspases, such as caspase-9 and caspase-8 [103]. Caspase-9 is responsible for initiating the intrinsic pathway, which is also referred to as the mitochondria-mediated pathway. On the other hand, caspase-8 is involved in the activation of the extrinsic apoptotic pathway, which is receptor-mediated [104]. Both pathways ultimately lead to the activation of executioner caspases, such as caspase-3 and caspase-6 [104]. Activation of caspase-3 and caspase-6, which are responsible for executing the process of cell death, results in the characteristic changes observed in apoptosis, such as the cleavage and fragmentation of DNA. This fragmentation of DNA plays a role in the degeneration of dopaminergic neurons in PD [104].

NEAT1 is a long non-coding RNA (lncRNA) that has been implicated in various cellular processes and diseases such as PD [105, 106]. Liu et al. reported that in the MPTP-treated mice, the number of TH^+^ cells (dopaminergic neurons) in the midbrain was significantly reduced compared to the control group. In the midbrain of mice with PD, the levels of NEAT1 and KLF4 were found to be elevated. This was accompanied by an increase in apoptosis-related proteins, including cleaved-caspase-3 and Bax, and a decrease in the expression of the anti-apoptotic protein Bcl-2. Similarly, in SH-SY5Y cells treated with the neurotoxin MPP + , the expression levels of NEAT1 and KLF4 were also increased in a dose-dependent manner. Similarly, the levels of apoptosis-related proteins were also altered in a dose-dependent manner. Knockdown of NEAT1 in MPP + -treated SH-SY5Y cells resulted in increased cell viability and decreased apoptosis. Moreover, the researchers discovered that KLF4 is a direct target of miR-124, and miR-124 has the ability to bind specifically to NEAT1. When KLF4 expression is increased, it counteracts the impact of NEAT1 knockdown on cell viability and apoptosis in SH-SY5Y cells treated with MPP + . In conclusion, this study proposes that NEAT1 could potentially facilitate apoptosis induced by MPTP/MPP + in PD by regulating the miR-124/KLF4 axis. Reducing the expression of NEAT1 improved cell survival and inhibited apoptosis, suggesting a possible therapeutic approach for PD [107].

miR-212 is a microRNA that has been shown to play a protective role in various neurological disorders, including PD [108]. Song et al. demonstrated that miR-212 exerts a neuroprotective effect against neuronal damage induced by PD. Their study revealed that miR-212 expression was reduced in SH-SY5Y cells treated with MPP + , which is commonly used as an in vitro model of PD. However, when miR-212 was overexpressed, it mitigated the detrimental effects of MPP + on SH-SY5Y cells. The experiment demonstrated that there was an increase in cell survival, a decrease in caspase-3 activity, LDH release, ROS production, TNF-α and IL-1β expression, as well as elevated SOD levels. The research also showed that miR-212 directly targets KLF4 and suppresses its expression. When KLF4 expression was restored in MPP + -induced SH-SY5Y cells, it reversed the protective effects of miR-212. Furthermore, it was discovered that the Notch signaling pathway plays a role in controlling the miR-212/KLF4 axis in MPP + -induced SH-SY5Y cells. To summarize, miR-212 reduces neuronal damage caused by MPP + in SH-SY5Y cells by targeting KLF4 and modulating the Notch signaling pathway [109]. These results indicate that targeting miR-212 could be a promising approach for treating PD.

MicroRNA-7 (miR-7) is a small RNA molecule that does not code for proteins and has been implicated in several biological processes, such as cell transformation, brain metastasis, growth regulation, and cancer progression [110, 111]. Kong et al. indicated that miR-7 plays a protective role in PD by preventing cell apoptosis. They used a human neuroblastoma cell line (SH-SY5Y) and treated it with MPP^+^, which is known to induce cell death in PD. The study demonstrated that when miR-7 levels were increased in SH-SY5Y cells, it resulted in enhanced cell viability and reduced cell apoptosis when exposed to MPP + treatment. Additionally, the research revealed that miR-7 directly targets KLF4. When KLF4 expression was reduced, it inhibited cell apoptosis, whereas overexpression of KLF4 negated the protective effect of miR-7 [112]. The results indicate that miR-7 provides protection against cell apoptosis caused by MPP + through its targeting of KLF4. Furthermore, KLF4, one of the Yamanaka transcription factors, has shown clinical relevance in PD [113]. While the clinical relevance of KLF4 in PD is promising, there are still limitations and challenges that need to be addressed. The long-term survival, functionality, and efficacy of the transplanted cells are still unknown [113].

Overall, KLF4 is a versatile transcription factor that plays important roles in various diseases. Its functions and effects can vary depending on the specific disease context and cellular environment. More investigation is required to gain a comprehensive understanding of the mechanisms involved in the functions of KLF4 and its potential as a target for therapeutic interventions in clinical settings.

## Conclusion

**Fig. 2:**
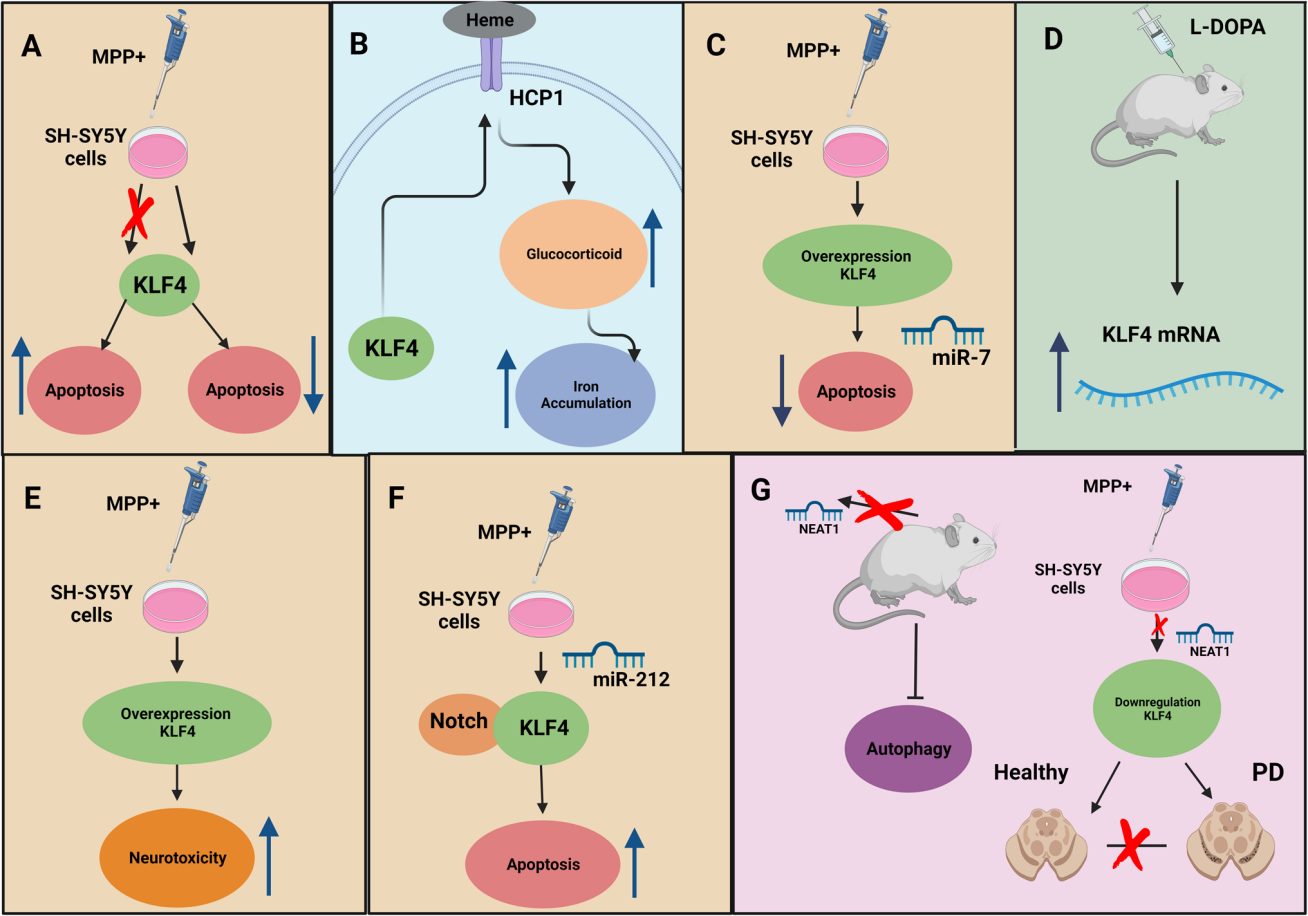
Potential KLF4-based strategies for treating PD. **A** KLF4 knockdown diminished cell death in MPP^+^-treated SH-SY5Y cells, while apoptosis was accelerated by overexpression KLF4. **B** In the KLF4-HCP1 signaling pathway, KLF4-mediated glucocorticoid-induces increases in the expression of HCP1, which causes iron accumulation. **C** Upregulation of KLF4 notably subsided the preservative impact of miR-7 on MPP^+^-induced apoptosis in SH-SY5Y cells. **D** KLF4 mRNA was induced by L-DOPA. **E** Upregulation of KLF4 increased the neurotoxicity of MPP^+^ in the form of rising cell vulnerability and oxidative stress. **F** miR-212-mediated protection impacts were abated after KLF4 expression restoration in MPP^+^-induced SH-SY5Y cells, represented as lessened cell viability and increased cell death. **G** NEAT1 knockdown can prevent MPTP-induced autophagy in vivo which lessens dopaminergic neuronal damage in a mouse model, and NEAT1 knockdown prevented the advent of PD by downregulating KLF4 in SH-SY5Y cells. HCP1, Heme Carrier Protein 1; MPP^+^, 1-methyl-4-phenylpyridinium; miR, miRNA; NEAT1, nuclear paraspeckle assembly transcript 1; KLF4, Krüppel-like factor 4

KLF4, as a transcription factor, regulates various cellular activities like cell growth, proliferation, and differentiation. Recently, concerns have arisen regarding the potential contribution of KLF4 to PD pathophysiology. Although the etiology of the disease is still obscure, the deregulation of the signaling pathways involved in cell survival and death, mitochondrial dysfunction, autophagy, oxidative stress, iron accumulation, and neuroinflammation are regarded to be involved in PD. In this review, we have collected relevant data. We discussed the modulating role of KLF4 on aberrant α-synuclein clearance, neuroinflammation, neuronal death, oxidative stress, and iron accumulation to elucidate the potential implication of KLF4 in PD pathogenesis. These findings support the idea that targeting KLF4 represents a prospective therapeutic strategy for PD also against deteriorated dopaminergic transmission in the brain (Fig. 2). However, limitations related to a low number of available data should be acknowledged. Given the context-dependent functions of KLF4, there is a gap in the knowledge of its role in PD pathogenesis. Thus, extensive research is required to understand the translational value of the KLF4-oriented therapeutic approach in PD.

## Data Availability

The literature analyzed during the current study is available at locations cited in the reference section.

## Abbreviations

CNS: Central nervous system
STAT3: Signal transducer and activator of transcription 3
Nrf2: Nuclear factor erythrocyte-associated factor 2
Trx 1: Thioredoxin-1
iPSCs: Induced pluripotent stem cells
MPTP: 1-Methyl-4-phenyl-1,2,3,6-tetrahydropyridine
MPP +: 1-Methyl-4-phenylpyridinium
6-OHDA: 6-Hydroxydopamine
Sp/Klf: Specificity protein/Krüppel-like factor
Aβ42: Amyloid β 42
BV2 cells: A unique type of microglial cells derived from C57/BL6 murine
EAE: Experimental autoimmune encephalomyelitis
MS: Multiple sclerosis
IL-1β: Interleukin-1β
iNOS: Inducible nitric oxide synthase
PI3K/AKT pathway: Phosphatidylinositol-3-kinase/protein kinase B pathway
MCP-1: Monocyte chemoattractant protein-1
Cox-2: Cyclooxygenase-2
NO: Nitric oxide
SNHG14: Small nucleolar RNA host gene 14
NF-kB: Nuclear factor kappa B
ROS: Reactive oxygen species
PINK1: PTEN-induced putative kinase 1
SNCA: Synuclein Alpha
LRRK2: Leucine-rich repeat kinase 2
GBA: Glucocerebrosidase
SOD1: Superoxide dismutase 1
LDH: Lactate dehydrogenase
NK1 receptor: Neurokinin-1 receptor
ERK5: Extracellular signal-regulated kinase 5
GSH: Glutathione
MDA: Malondialdehyde
NEAT1: Nuclear-enriched abundant transcript 1
Bcl2: B-cell lymphoma 2
Bax: Bcl-2-associated X protein
